# Prediction and Analysis of Creep Rupture Life of 9Cr Martensitic-Ferritic Heat-Resistant Steel by Neural Networks

**DOI:** 10.3390/ma19020257

**Published:** 2026-01-08

**Authors:** Muhammad Ishtiaq, Seungmin Hwang, Won-Seok Bang, Sung-Gyu Kang, Nagireddy Gari Subba Reddy

**Affiliations:** 1 Department of Materials Engineering and Convergence Technology, Gyeongsang National University, 501, Jinju 52828, Republic of Korea; ishtiaq145@gnu.ac.kr (M.I.); smh@gnu.ac.kr (S.H.); 2 Department of Business Administration, Entrepreneurship Research Institute, Gyeongsang National University, 501, Jinju 52828, Republic of Korea

**Keywords:** 9Cr heat-resistant steel, artificial neural network, creep rupture, prediction, quantitative estimation

## Abstract

Thermal and nuclear power systems require materials capable of sustaining high mechanical and thermal loads over prolonged service durations. Among these, 9Cr heat-resistant steels are particularly attractive due to their superior mechanical strength and extended creep rupture life, making them suitable for extreme environments. In this study, multiple machine learning models were explored to predict the creep rupture life of 9Cr heat-resistant steels. A comprehensive dataset of 913 samples, compiled from experimental results and literature, included eight input variables—covering chemical composition, stress, and temperature—and one output variable, the creep rupture life. The optimized artificial neural network (ANN) model achieved the highest predictive accuracy with a regularization coefficient of 0.01, 10,000 training iterations, and five hidden layers with 30 neurons per layer, attaining an R^2^ of 0.9718 for the test dataset. Beyond accurate prediction, single- and two-variable sensitivity analyses were used to elucidate statistically meaningful trends and interactions among the input parameters governing creep rupture life. The analyses indicated that among all variables, test conditions—particularly the test temperature—exert a pronounced negative effect on creep life, significantly reducing durability at elevated temperatures. Additionally, an optimization module enables identification of input conditions to achieve desired creep life, while the Index of Relative Importance (I_RI_) and quantitative effect analysis enhance interpretability. This framework represents a robust and reliable tool for long-term creep life assessment and the design of 9Cr steels for high-temperature applications.

## 1. Introduction

The ever-growing global demand for power generation requires energy systems to operate at elevated temperatures for extended periods to achieve higher efficiency, reduce greenhouse gas emissions, and minimize energy shortages [1]. Consequently, the materials used in boilers, heaters, and piping systems must exhibit exceptional high-temperature strength and structural stability [2]. Among these, 9Cr steels have emerged as a key structural material in power plants owing to their excellent creep resistance, prolonged service life, and superior thermal stability [3]. The creep performance and long-term durability of these steels are strongly influenced by their chemical composition, alloying elements, and the applied heat treatment conditions, which collectively determine their microstructural characteristics and creep properties [4]. Many components fabricated from such steels are now approaching their designed service lifetimes and exhibit varying degrees of creep damage. However, in many cases, these systems still retain substantial residual life. Conventional creep evaluation approaches face major difficulties in validating material performance and predicting service life because they require lengthy tests to capture the full extent of damage evolution [5]. Therefore, accurately predicting the creep rupture life of these components is essential not only for ensuring operational safety and reliability but also for optimizing maintenance schedules and supporting sustainable power generation practices.

Various constitutive and parameter-based models and equations have been reported for predicting and analyzing the creep behavior of 9Cr steels. N. Eberle and F.L. Jones [6] applied the θ projection concept of Evans and Wilshire to modified 9Cr–1Mo steel and found that, although the model effectively represented the general shape of creep curves and accurately predicted short-term behavior, it failed to precisely capture the temperature and stress dependencies of the parameters, resulting in less reliable long-term creep predictions. R. Oruganti et al. [7] employed damage mechanics–based creep models for 9Cr steels and revealed that the creep behavior is predominantly governed by key microstructural features. W. Bendick et al. [8] employed a time–temperature parameter approach to evaluate the creep rupture strength of P91 steel and concluded that the ISO CRD method provided the most accurate overall fit to the experimental data. W. G. Kim et al. [9] developed and compared three creep constitutive models to predict the long-term creep behavior and life of Gr. 91 steel. Their analysis, performed using nonlinear least-squares fitting of experimental data at 600 °C, revealed that the combined exponential and omega (CEO) model provided the most accurate prediction among the three approaches—namely, the combined power-law and omega (CPO) model and the combined logarithmic and omega (CLO) model. However, the predictive accuracy of these models varied with applied stress: the CEO model performed better in the low-stress regime (≈160 MPa), whereas the CPO model showed superior agreement with experimental results at higher stresses (>160 MPa). M. Basirat et al. [10] employed continuum damage modeling (CDM) to investigate the creep behavior and damage evolution in 9Cr steels.

Since the emergence of machine learning (ML) and its integration into materials science and engineering, Artificial Neural Network (ANN)-based models have been widely employed to predict various material properties, including mechanical behavior [11], thermal properties [12], corrosion resistance [13], and creep performance [14] of structural alloys. In particular, numerous studies have reported the use of different ML-based frameworks to estimate the creep properties of 9Cr steels, owing to their critical role in high-temperature power plant applications [15]. B.O. Kong. et al. [16] proposed the extra gradient boosting (XGB) regressor model to predict the creep life of 9Cr steel and, through SHAP analysis, demonstrated that normalization temperature is the most influential factor, followed by various alloying elements. Mamun et al. [17] developed a machine learning–augmented predictive and generative framework for estimating the rupture life of 9Cr steel, where a GB model was effectively utilized to accurately predict the creep rupture life. Y. Tan et al. [18] compared the predictive accuracy of five different ML models for estimating the creep lifetime of 9Cr steels using data obtained from the European Creep Collaborative Committee (ECCC).

Previously reported models were generally limited in scope, as they are either applicable only within specific stress ranges or tailored to particular alloy compositions, while others are quite complex and require extensive programming and calculations, making them less practical for routine applications. To address these limitations, we developed an ANN model to predict the creep life of 9Cr steels using a comprehensive dataset comprising 926 samples with varying chemical compositions and heat treatment parameters. The model was further analyzed through single-variable and two-variable sensitivity analyses to evaluate the individual and combined effects of key parameters on creep life. Additionally, we introduced the Index of Relative Importance (I_RI_) to quantitatively assess the influence of each alloying element. To enhance practical applicability, a user-friendly graphical user interface (GUI) was also developed, enabling easy use of the model for industrial prediction and optimization purposes.

## 2. Materials and Methods

### 2.1. Data Collection

A comprehensive dataset comprising 927 data points was collected from various published sources on 9Cr heat-resistant steels [8,19,20]. The dataset includes detailed information on chemical compositions (including minor alloying elements), heat treatment conditions, testing parameters, and corresponding creep rupture life data. The typical compositional range of the steels considered in this dataset is given in Table 1.

### 2.2. Data Preprocessing and Cleaning

During data preprocessing, outlier entries were identified and removed to enhance the consistency and reliability of the model inputs. Certain alloying elements—namely sulfur (~0.02 wt%), aluminum (~0.009 wt%), boron (~0.0003 wt%), tantalum (~0.0003 wt%), and oxygen (~0.01 wt%)—exhibited negligible variation across the dataset. Preliminary single- and two-variable sensitivity analyses confirmed that variations in these elements had no statistically discernible influence on the predicted creep rupture life, justifying their exclusion to reduce model dimensionality and avoid noise. Additionally, the cooling medium after annealing was excluded, as it is conventionally understood to involve furnace cooling. Since these parameters remained nearly constant and were not expected to influence the model’s predictive performance, they were omitted from further analysis. Following data cleaning and refinement, the final dataset comprised 913 valid entries, which were utilized for model development. The statistical details of the cleaned dataset, including the minimum, maximum, mean, and standard deviation values for each parameter, are presented in Table 2.

### 2.3. Distribution of Parameters

**Figure 1:**
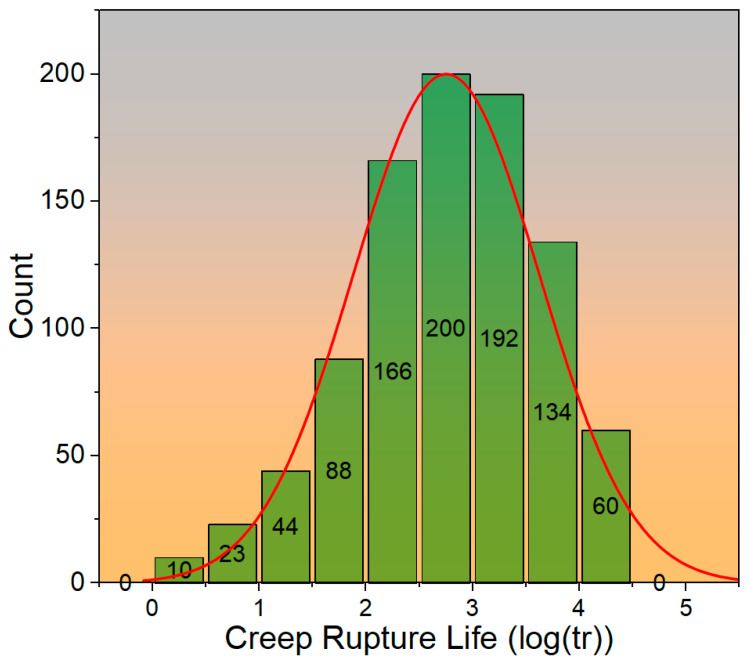
The frequency distribution histogram of the dataset used for the development and evaluation of the ANN model.

Figure 1 presents a histogram illustrating the distribution of the dataset used for the development and evaluation of the ANN model. The *x*-axis represents the binned values od creep rupture life (log(tr)), while the *y*-axis indicates the frequency or count of data points within each bin. A total of nine bins are displayed, with counts ranging from 0 to 200. The overlaid red curve represents a fitted probability density function, providing a visual approximation of the underlying distribution. From the histogram, it is evident that the dataset exhibits a roughly unimodal distribution, with the highest concentration of data points occurring between approximately 2.5 to 3.5. Lower and higher bins show a decreasing frequency, indicating fewer instances at the extremes. The presence of this near-normal distribution suggests that the dataset is reasonably balanced, with minimal skewness, which is favorable for training a predictive model.

### 2.4. Pairwise Correlation Analysis

**Figure 2:**
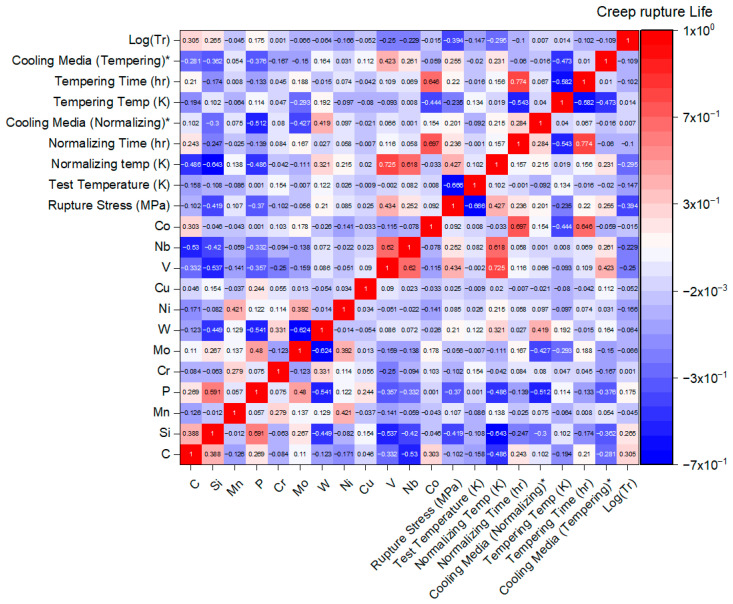
The heatmap showing the pairwise correlation between all variables. The strength and direction of correlation are represented by the color intensity. The numerical values within each cell indicate the correlation coefficient, ranging from –1 to 1. * (0 for furnace cooling, 1 for air cooling, 2 for oil quenching, and, 3 for water quenching).

To illustrate the pairwise correlations among all variables in the dataset, a heatmap is presented in Figure 2, showing the relationships between chemical composition, heat treatment parameters, and the creep rupture life of 9Cr heat-resistant steels. This visualization provides an initial overview of how the input parameters interact and serves as a guide for developing the ANN model. The color intensity in the heatmap indicates the strength of the correlation between each pair of variables, while the numerical values displayed within each cell quantify the correlation coefficient, ranging from −1 to 1. Positive values indicate a direct (proportional) relationship, whereas negative values indicate an inverse relationship between the variables.

For example, carbon (C) content exhibits a positive correlation of 0.305 with the creep rupture life (log tr), represented by light red, indicating that higher C levels tend to enhance creep resistance. Similarly, silicon (Si) shows a positive correlation of 0.265, as both C and Si are carbide-forming elements that can precipitate stable carbides, thereby delaying rupture during creep. In contrast, the applied stress demonstrates a strong negative correlation of −0.394 with log (tr) represented by light blue, indicating that an increase in stress reduces creep rupture life. The diagonal cells of the heatmap show the correlation of each parameter with itself, which is naturally 1 and represented by red, indicating a perfect positive correlation. Although this heatmap provides valuable insights into the initial relationships among variables, the interactions are highly complex due to the large number of parameters involved. The trained ANN model is therefore essential, as it can capture these intricate interactions and accurately predict the contribution of each variable to the creep rupture life.

## 3. Methodology

### 3.1. Workflow of the Study

The present study aimed to develop an ANN model for predicting the creep rupture life of 9Cr heat-resistant steels. The objectives include evaluating the effect of individual input variables, understanding the combined influence of multiple variables, quantitatively estimating the contribution of each parameter, and enabling virtual steel design with maximized creep rupture life. Additionally, the study seeks to identify optimal heat treatment routes to achieve improved microstructural stability and sustained load-bearing capacity, as well as to recommend testing conditions suitable for enhancing creep performance.

**Figure 3:**
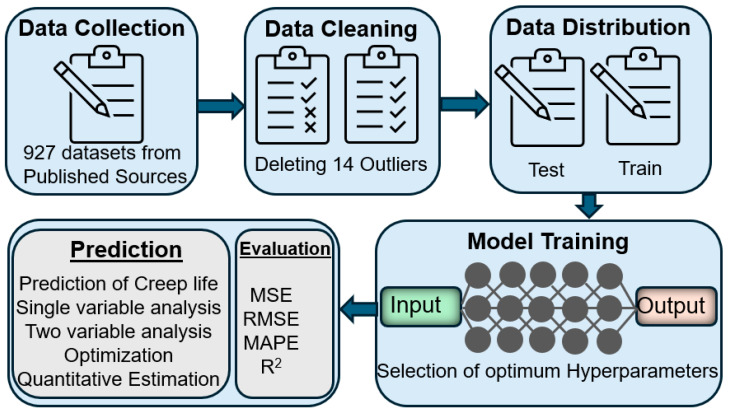
Workflow for the development of the ANN model to predict the creep rupture life of 9Cr heat-resistant steels.

Figure 3 illustrates the overall workflow for developing the ANN model to predict creep rupture life. The process begins with data collection, followed by preprocessing and cleaning to eliminate outliers. The finalized dataset was randomly shuffled and then divided into training and testing subsets using a well-established 80:20 split. Out of total 913 datasets, 730 data points were used for model training and 183 data points were reserved for independent testing and performance evaluation. No stratification by stress level was applied during the splitting process; instead, random sampling was used to ensure that the overall statistical distribution of input variables, including stress, temperature, and composition, was reasonably preserved across both subsets. Next, optimal hyperparameters were selected to ensure robust model performance and accurate prediction of creep behavior under diverse conditions.

### 3.2. ANN Model Development

An ANN typically comprises a series of structured layers, including an input layer representing the input variables, one or more hidden layers, and an output layer that provides the predicted properties. Each layer contains a set of neurons that compute a weighted sum of the outputs from the preceding layer. During training, these weights are iteratively adjusted to minimize the prediction error, and the process continues until the model achieves a satisfactory level of accuracy. The hyperparameters—such as the number of neurons, regularization, and number of training iterations—are carefully optimized to enhance prediction precision. In the present study, the ANN was trained using the backpropagation algorithm with a sigmoid transfer function, implemented in the C programming language.

### 3.3. Selection of Hyperparameters Based on Training Behavior of Model

**Figure 4:**
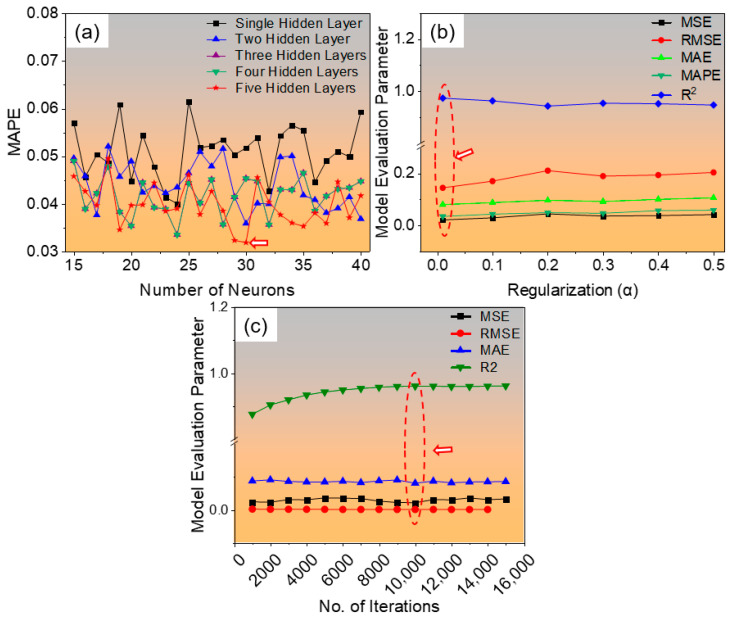
Optimization of the developed ANN model through systematic variation in key hyperparameters: (**a**) effect of the number of hidden layers and neurons on MAPE, (**b**) influence of the regularization coefficient on model accuracy, and (**c**) variation in performance metrics (R^2^, MSE, RMSE, MAE, and MAPE) with the number of training iterations. The optimal configurations are indicated by arrows.

The developed ANN model was systematically trained by varying the number of neurons and hidden layers to optimize prediction accuracy. Initially, models with single, double, triple, quadruple, and quintuple hidden layers were evaluated, with the number of neurons in each layer varied from 15 to 40. The mean absolute percentage error (MAPE) corresponding to each configuration is presented in Figure 4a. It was observed that the network architecture comprising five hidden layers with 30 neurons per layer exhibited the lowest error and highest prediction accuracy, yielding a MAPE of 0.031896 for the training dataset. Hence, this configuration was selected as the optimal architecture. Subsequently, the effect of regularization was examined by varying the regularization coefficient from 0.01 to 0.5, as illustrated in Figure 4b. The results revealed that a regularization value of 0.01 produced the best performance, achieving a coefficient of determination (R^2^) of 0.9732, along with the lowest values of the MSE (0.020799), RMSE (0.144218), MAE (0.07980), and MAPE (0.0341). Finally, the influence of the number of training iterations was evaluated by varying it from 1000 to 15,000, as shown in Figure 4c. The model trained with 10,000 iterations demonstrated the highest efficiency, attaining R^2^ = 0.9606, MSE = 0.0208, RMSE = 0.000351, and MAE = 0.07980. The optimal conditions for the number of neurons, hidden layers, regularization, and iterations are indicated by arrows in Figure 4.

**Figure 5:**
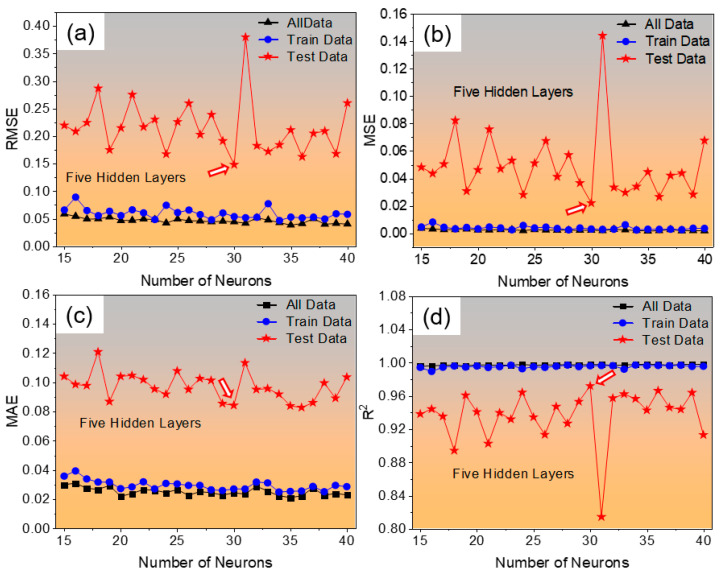
Effect of the number of neurons on the predictive performance of the developed ANN model in terms of (**a**) root mean squared error (MSE), (**b**) mean squared error (RMSE), (**c**) mean absolute error (MAE), and (**d**) coefficient of determination (R^2^) for all, training, and test datasets. The configuration with five hidden layers and 30 neurons exhibited the lowest error values and highest accuracy. The arrows point to the number of neurons that yield the best model performance.

After optimizing the ANN architecture through systematic hyperparameter tuning, the configuration comprising five hidden layers with 30 neurons in each layer was identified as the most accurate and efficient structure. To further assess the model’s predictive reliability, the influence of the number of neurons on model accuracy was evaluated using four key statistical indicators: MSE, RMSE, MAE, and R^2^. The results for these metrics are illustrated in Figure 5a–d, corresponding to all data, training data, and test data, respectively. As shown in the figure, the model with 30 neurons in five hidden layers achieved the best performance, demonstrating a minimum MSE of 0.0218, RMSE of 0.1478, and MAE of 0.0841, along with a high coefficient of determination (R^2^) of 0.9718 for the test dataset. These results confirm that this neural configuration effectively balances bias and variance, providing high predictive accuracy and strong generalization capability across both training and unseen test datasets.

### 3.4. Graphical User Interface (GUI)

**Figure 6:**
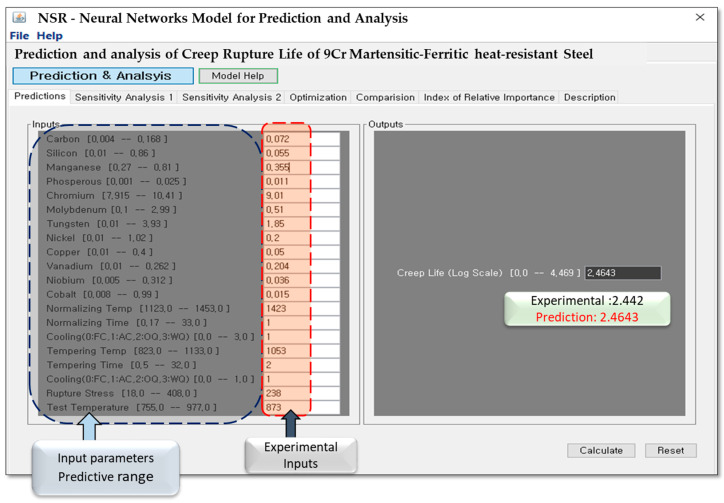
Graphical User Interface (GUI) of the developed ANN model for predicting the creep rupture life (log tr) of 9Cr heat-resistant steels.

A practical Graphical User Interface (GUI) was developed using Java 1.4 to make the ANN model easy to use on any computer system. As shown in Figure 6, the interface allows the user to enter all required inputs—chemical composition, normalizing and tempering conditions, cooling mode, rupture stress, and test temperature. Each input is displayed with its allowable range so that users can quickly check whether the selected values are realistic for 9Cr heat-resistant steels. After the values are entered, the model immediately calculates the creep rupture life in log scale and displays both the predicted and experimental values side by side for quick comparison.

The GUI also includes two built-in sensitivity analysis tools. The Single-Variable module shows how the creep life changes when one parameter is varied, while the Two-Variable module examines the combined influence of any two input variables. Even though the ANN was trained only with numerical data, the model still captures important metallurgical effects that control creep behavior. Overall, this GUI works as a practical engineering tool that helps users evaluate creep performance, compare different alloy or heat-treatment options, and make faster design decisions. Moreover, the GUI includes an optimization function, allowing users to identify the optimal steel composition, heat treatment, or testing condition required to achieve a target creep life. The Index of Relative Importance (I_RI_) module provides a quantitative assessment of each parameter’s influence on creep performance. All these aspects are elaborated upon in the subsequent results section.

## 4. Results

### 4.1. Model Prediction Results

**Figure 7:**
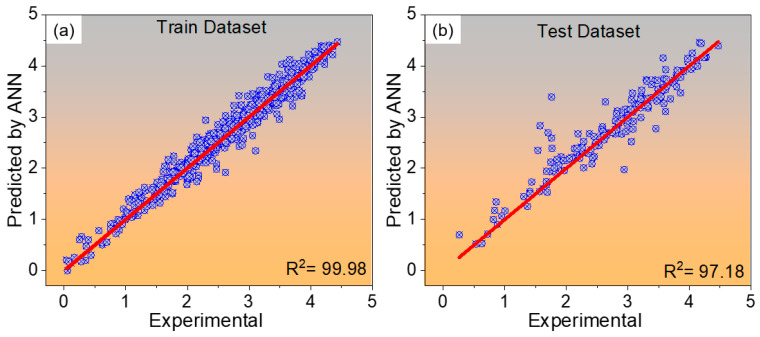
Parity plots comparing the ANN-predicted and experimentally measured creep rupture life values for 9Cr heat-resistant steels. (**a**) Training dataset and (**b**) test dataset. The red line represents the ideal 1:1 correlation, indicating perfect agreement between predicted and experimental values.

The predictive performance of the optimized ANN architecture is illustrated in Figure 7, where the parity plots compare the ANN-predicted and experimentally measured creep rupture life values for 9Cr heat-resistant steels. The results for the training and testing datasets are shown in Figure 7a,b, respectively. The close clustering of data points around the red 1:1 parity line indicates a strong correlation between the predicted and experimental values, confirming the model’s capability to capture the underlying relationships effectively. The high coefficients of determination (R^2^ = 99.98% for training and 97.18% for testing) further validate the reliability, accuracy, and generalization performance of the optimized ANN model.

### 4.2. Single Variable Sensitivity Analysis

**Figure 8:**
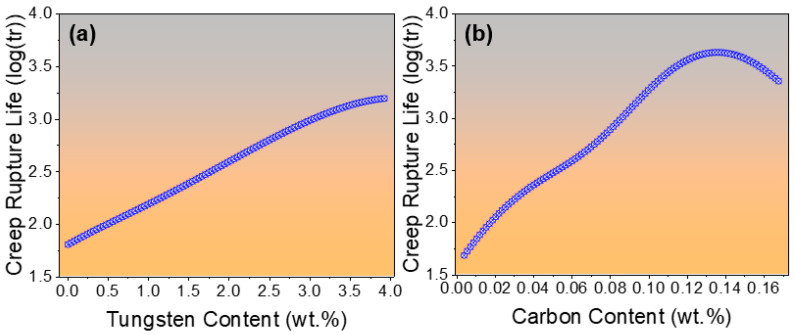
Effect of alloying elements on the creep rupture life of 9Cr heat-resistant steel; (**a**) Effect of tungsten content, (**b**) effect of carbon content.

The influence of individual variables, whether related to chemical composition or heat treatment parameters, on creep rupture life is complex and non-linear. To quantify the effect of a single variable, a single-variable sensitivity analysis can be employed, providing insight into how changes in that parameter impact the final creep rupture life of 9Cr heat-resistant steel. For instance, Figure 8a illustrates the effect of tungsten (W), one of the key alloying elements, on creep rupture life. The analysis shows that increasing W content enhances the creep rupture life, which can be attributed to the improved high-temperature strength provided by W. This improvement is attributed to the dislocation pinning effect induced by the formation of stable M_23_C_6_ carbides, which precipitate through the interaction of tungsten with carbon. These carbides effectively restrict dislocation motion and enhance the high-temperature strength of 9Cr steels [21]. Furthermore, the tempering treatment contributes to delaying dislocation recovery, as the stabilized carbide precipitates lower the self-diffusivity of iron, thereby improving creep resistance [22].

As shown in Figure 8b, the effect of C content on creep rupture life follows a non-linear trend: an increase in C initially enhances creep rupture life by promoting the formation of stable carbides that strengthen the microstructure. However, beyond an optimal level, excess C leads to the formation of brittle and coarse carbides, which may act as crack initiation sites along grain boundaries, resulting in a reduction in creep rupture life. This observation aligns with previously reported studies, where excessive carbide precipitation was found to degrade creep ductility and accelerate crack propagation in 9Cr heat-resistant steels [23]. These metallurgical observations have been experimentally validated in previous studies [22], which reported that 9Cr heat-resistant steels containing W exhibit a higher carbide density and more uniform distribution compared to W-free steels when tempered at 800 °C. Although such microstructural information and metallurgical insights were not explicitly provided to the ANN model, the model is capable of implicitly capturing and predicting the enhanced creep rupture life of W-containing steels, effectively mimicking the beneficial effects of W addition.

### 4.3. Two Variables Sensitivity Analysis

**Figure 9:**
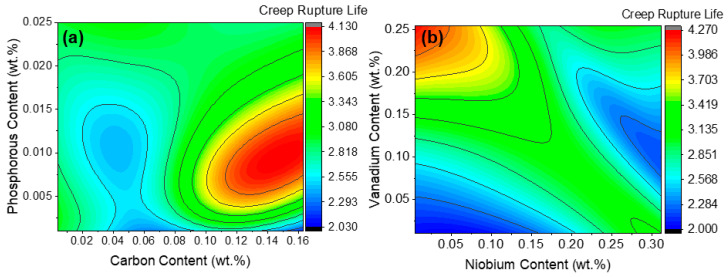
Contour plots illustrating the ANN-predicted combined effects of (**a**) carbon and phosphorus, and (**b**) niobium and vanadium, on the creep rupture life of 9Cr heat-resistant steel.

The combined influence of alloying elements on creep rupture life is inherently complex due to intricate metallurgical interactions and thermally activated diffusion processes. For example, the interaction between C and P in 9Cr heat-resistant steels can have competing effects on microstructural stability. Carbon enhances creep strength by promoting M_23_C_6_ carbide precipitation along prior austenite and lath boundaries, which impedes dislocation motion and recovery [24]. However, phosphorus tends to segregate at grain boundaries, reducing cohesion and promoting intergranular embrittlement during long-term exposure [25]. When both elements are present at higher levels, carbides act as preferential segregation sites for P, intensifying boundary weakening. Thus, steels with higher C and P contents exhibit shorter creep rupture life compared to those with moderate C and lower P, as shown in Figure 9a.

Similarly, the contour plot in Figure 9b demonstrates that higher V combined with lower Nb levels yields superior creep life, while higher Nb alone does not [26]. This is attributed to V forming a stable Z-phase that effectively pins dislocations and hinders grain boundary sliding [27], whereas excessive Nb promotes coarser NbC precipitates with lower thermal stability, reducing long-term creep resistance [28]. The formation of the Z-phase due to V addition has been experimentally validated through TEM analysis in previous studies [29]. It has been reported that in V-free steels, only MX-type precipitates are present, whereas the addition of approximately 0.3 wt.% V leads to the formation of a new type of precipitate—the Z-phase—after prolonged exposure at 700 °C.

### 4.4. Index of Relative Importance and Quantitative Estimation

**Figure 10:**
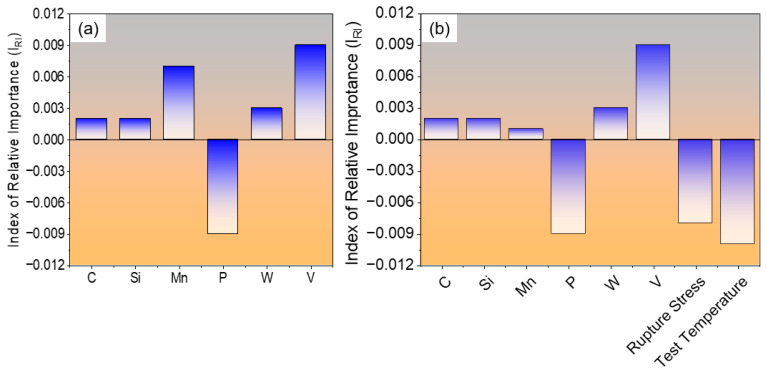
Bar graphs showing the quantitative effect by index of relative importance for a specific 9Cr heat-resistant steel; (**a**) effect of certain alloying elements, (**b**) effect of test conditions along with alloying elements.

The qualitative and quantitative influence of input parameters on the creep rupture life of 9Cr heat-resistant steels can be effectively predicted using the developed ANN model. The Index of Relative Importance (I_RI_) for selected alloying elements is presented in Figure 10a, illustrating their contribution to creep performance for a specific steel composition: 0.063C–0.360Si–0.650Mn–0.017P–9.04Cr–2.99Mo–0.01W–1.02Ni–0.01V–0.005Nb –0.015Co, normalized at 1338 °C for 30 min and subsequently tempered at 987 °C for 1 h, with creep testing conducted at 241 MPa and 866 °C. For this composition, the ANN model predicts that all alloying elements contribute positively to creep rupture life except phosphorus, which exhibits a negative effect. As mentioned earlier, P can segregate at grain boundaries and reduce cohesion to promote intergranular embrittlement during prolonged exposure at elevated temperatures, thereby decreasing creep life [25].

To further evaluate the capability of the ANN model in capturing the qualitative effects of test conditions on creep rupture life, test temperature, and applied rupture stress were included as input variables. The model predicted a negative influence of both parameters on creep life, indicating that higher test temperatures or increased applied stress lead to a reduction in the creep rupture life of 9Cr heat-resistant steels, as illustrated in Figure 10b. These predictions are consistent with metallurgical understanding, as elevated temperatures accelerate diffusion and dislocation recovery, while higher stresses enhance creep strain rates, both contributing to earlier failure [27,30].

To further quantify the effect of individual variables, all input parameters were initially set to their minimum values (0.004 C–0.01 Si–0.27 Mn–0.001 P–7.915 Cr–0.1 Mo–0.01 Ni–0.01 Cu–0.01 V–0.005 Nb–0.08 Co), normalized at 1123 °C for 0.17 h, tempered at 823 °C for 0.5 h, and creep tested under 18 MPa at 755 °C. Under these conditions, the ANN model predicted a creep rupture life of 5.0276 h. Each input variable was then incrementally increased individually to evaluate its quantitative impact on creep life. The resulting incremental or decremental effects for each parameter are visualized in Table 3. Finally, the input variables were set to match one of the experimental steel compositions and corresponding test conditions, allowing for a direct comparison between predicted and experimental creep rupture life. The experimental value of log(tr) is 2.442, while the ANN model predicted 2.4643, resulting in a percentage error of approximately 0.91%. This close agreement confirms the accuracy and reliability of the ANN model in capturing the effects of alloying elements and processing parameters on creep performance.

From the quantitative analysis, it is evident that variations in chemical composition have a relatively minor effect on the final creep rupture life, as the changes are limited and all samples belong to the 9Cr heat-resistant steel class. In contrast, the major influencing factors are the applied rupture stress and, in particular, the test temperature, both of which exert a negative effect on creep life, with higher values leading to a significant reduction in creep rupture life.

## 5. Conclusions

From the present study, the following conclusions can be drawn:

1. An artificial neural network (ANN) model was successfully developed to predict the creep rupture life of 9Cr heat-resistant steels using a dataset of 913 samples, including chemical composition, heat treatment parameters, stress, and temperature.
2. The optimized ANN architecture, comprising five hidden layers with 30 neurons each, a regularization coefficient of 0.01, and 10,000 training iterations, achieved the highest predictive accuracy with an R^2^ of 0.9718 for the test dataset and low error metrics, demonstrating excellent reliability and generalization.
3. Single-variable and two-variable sensitivity analyses revealed the individual and combined effects of alloying elements and processing parameters on creep rupture life, highlighting both beneficial (e.g., carbon, vanadium) and detrimental (e.g., phosphorus) influences.
4. The Index of Relative Importance (I_RI_) and quantitative effect analysis allowed for the ranking of input variables based on their impact on creep rupture life, enhancing the interpretability of the model.
5. The creep rupture life of 9Cr heat-resistant steel is primarily governed by test conditions—particularly applied stress and temperature—while variations in chemical composition have a comparatively minor effect.
6. The developed ANN model was implemented in a user-friendly graphical interface (GUI), enabling predictions for infinite input combinations, optimization of steel composition and processing parameters, and assessment of metallurgical trends without prior explicit knowledge.

Overall, this framework provides a powerful tool for the design, optimization, and long-term assessment of 9Cr heat-resistant steels for high-temperature applications in thermal and nuclear power systems.

**Limitations and Future work:** While the present study demonstrates the capability of the ANN framework to predict creep rupture life and capture statistically meaningful trends, the analysis is limited to a single machine-learning architecture. Future studies will focus on a systematic comparison with other modern machine-learning algorithms (e.g., Random Forest, XGBoost) as well as physics-based or hybrid modeling approaches.

## Figures and Tables

**Table 1:** The typical chemical composition (wt.%) of the 9Cr heat-resistant steel.

C	Si	Mn	P	Cr	Mo	W	Ni	Cu	V	Nb	Co
0.004–0.17	0.01–0.86	0.27–0.81	0.001–0.025	7.9–10.4	0.1–2.99	0.01–3.93	0.01–1.02	0.01–0.4	0.01–0.26	0.005–0.31	0.008–0.99

**Table 2:** Statistical summary of the cleaned dataset, showing the minimum, maximum, mean, and standard deviation values for the key parameters used in model development.

Data	Feature	Minimum	Maximum	Mean	Std. Deviation
Input	C	0.004	0.168	0.089	0.028
	Si	0.01	0.86	0.265	0.196
	Mn	0.27	0.81	0.448	0.074
	P	0.001	0.025	0.010	0.006
	Cr	7.915	10.41	8.833	0.304
	Mo	0.1	2.99	0.859	0.393
	W	0.01	3.93	0.485	0.823
	Ni	0.01	1.02	0.143	0.140
	Cu	0.01	0.4	0.056	0.063
	V	0.01	0.262	0.152	0.088
	Nb	0.005	0.312	0.057	0.053
	Co	0.008	0.99	0.061	0.165
	Normalizing Temperature (K)	1123	1453	1306.61	70.61
	Normalizing Time (Hr)	0.17	33	1.54	3.20
	Cooling Media (Normalizing) *	-	-	-	-
	Tempering Temperature (K)	823	1133	1029.06	51.96
	Tempering Time (Hr)	0.5	32	2.49	4.95
	Cooling Media (Tempering) *	-	-	-	-
	Rupture Stress (MPa)	18	408	139.78	71.39
	Test Temperature (K)	755	977	894.47	48.26
Output	Creep Life (Log (tr))	0	4.469	2.756	0.864

* (0 for furnace cooling, 1 for air cooling, 2 for oil quenching, and, 3 for water quenching).

**Table 3:** The stepping variation in the creep rupture life of 9CR heat-resistant steel by the quantitative addition of the input variable.

Steel Composition, Heat Treatment Parameters, and Test Conditions	Log(tr)	Variation
0.004C–0.01Si–0.27Mn–0.001P–7.915Cr–0.1Mo–0.01W–0.01Ni–0.01Cu–0.01V–0.005Nb–0.008Co, normalized at 1123 °C for 0.17 hrs, tempered at 823 °C for 0.5 hrs, tested under 18MPa at 755 °C	5.0273	-
0.039C–0.01Si–0.27Mn–0.001P–7.915Cr–0.1Mo–0.01W–0.01Ni–0.01Cu–0.01V–0.005Nb–0.008Co, normalized at 1123 °C for 0.17 hrs, tempered at 823 °C for 0.5 hrs, tested under 18MPa at 755 °C	5.0275	0.0002
0.039C–0.044Si–0.27Mn–0.001P–7.915Cr–0.1Mo–0.01W–0.01Ni–0.01Cu–0.01V–0.005Nb–0.008Co, normalized at 1123 °C for 0.17 hrs, tempered at 823 °C for 0.5 hrs, tested under 18MPa at 755 °C	5.0279	0.0004
0.039 C–0.044 Si–0.49Mn–0.001 P–7.915Cr–0.1Mo–0.01W–0.01Ni–0.01Cu–0.01V–0.005Nb–0.008Co, normalized at 1123 °C for 0.17 hrs, tempered at 823 °C for 0.5 hrs, tested under 18MPa at 755 °C	5.0270	−0.0009
0.039C–0.044Si–0.49Mn–0.011P–7.915Cr–0.1Mo–0.01W–0.01Ni–0.01Cu–0.01V–0.005Nb–0.008Co, normalized at 1123 °C for 0.17 hrs, tempered at 823 °C for 0.5 hrs, tested under 18MPa at 755 °C	5.0273	0.0003
0.039C–0.044Si–0.49Mn–0.011P–9.01Cr–0.1Mo–0.01W–0.01Ni–0.01Cu–0.01V–0.005Nb–0.008Co, normalized at 1123 °C for 0.17 hrs, tempered at 823 °C for 0.5 hrs, tested under 18MPa at 755 °C	5.0276	0.0003
0.039C–0.044Si–0.49Mn–0.011P–9.01Cr–0.51Mo–0.01W–0.01Ni–0.01Cu–0.01V–0.005Nb–0.008Co, normalized at 1123 °C for 0.17 hrs, tempered at 823 °C for 0.5 hrs, tested under 18MPa at 755 °C	5.0276	0.0000
0.039C–0.044Si–0.49Mn–0.011P–9.01Cr–0.51Mo–1.85W–0.01Ni–0.01Cu–0.01V–0.005Nb–0.008Co, normalized at 1123 °C for 0.17 hrs, tempered at 823 °C for 0.5 hrs, tested under 18MPa at 755 °C	5.0273	−0.0003
0.039C–0.044Si–0.49Mn–0.011P–9.01Cr–0.51Mo–1.85W–0.01Ni–0.01Cu–0.01V–0.005Nb–0.008Co, normalized at 1123 °C for 0.17 hrs, tempered at 823 °C for 0.5 hrs, tested under 18MPa at 755 °C	5.0269	−0.0004
0.039C–0.044Si–0.49Mn–0.011P–9.01Cr–0.51Mo–1.85W–0.2Ni–0.01Cu–0.01V–0.005Nb–0.008Co, normalized at 1123 °C for 0.17 hrs, tempered at 823 °C for 0.5 hrs, tested under 18MPa at 755 °C	5.0270	0.0001
0.039C–0.044Si–0.49Mn–0.011P–9.01Cr–0.51Mo–1.85W–0.01Ni–0.2Cu–0.01V–0.005Nb–0.008Co, normalized at 1123 °C for 0.17 hrs, tempered at 823 °C for 0.5 hrs, tested under 18MPa at 755 °C	5.014	0.013
0.039C–0.044Si–0.49Mn–0.011P–9.01Cr–0.51Mo–1.85W–0.01Ni–0.2Cu–0.204V–0.005Nb–0.008Co, normalized at 1123 °C for 0.17 hrs, tempered at 823 °C for 0.5 hrs, tested under 18MPa at 755 °C	5.0275	0.0135
0.039C–0.044Si–0.49Mn–0.011P–9.01Cr–0.51Mo–1.85W–0.01Ni–0.01Cu–0.01V–0.036Nb–0.008Co, normalized at 1123 °C for 0.17 hrs, tempered at 823 °C for 0.5 hrs, tested under 18MPa at 755 °C	5.0275	0.000
0.039C–0.044Si–0.49Mn–0.011P–9.01Cr–0.51Mo–1.85W–0.01Ni–0.01Cu–0.01V–0.005Nb–0.015Co, normalized at 1123 °C for 0.17 hrs, tempered at 823 °C for 0.5 hrs, tested under 18MPa at 755 °C	5.0275	0.000
0.039C–0.044Si–0.49Mn–0.011P–9.01Cr–0.51Mo–1.85W–0.01Ni–0.01Cu–0.01V–0.005Nb–0.015Co, normalized at 1423 °C for 1 h, tempered at 823 °C for 0.5 hrs, tested under 18MPa at 755 °C	5.0275	0.000
0.039C–0.044Si–0.49Mn–0.011P–9.01Cr–0.51Mo–1.85W–0.01Ni–0.01Cu–0.01V–0.005Nb–0.015Co, normalized at 1123 °C for 1 hrs, tempered at 1053 °C for 2 h, tested under 18MPa at 755 °C	5.0272	−0.0003
0.039C–0.044Si–0.49Mn–0.011P–9.01Cr–0.51Mo–1.85W–0.01Ni–0.01Cu–0.01V–0.005Nb–0.015Co, normalized at 1123 °C for 1 hrs, tempered at 1053 °C for 2 hrs, tested under 238MPa at 755 °C	5.0089	0.0017
0.039C–0.044Si–0.49Mn–0.011P–9.01Cr–0.51Mo–1.85W–0.01Ni–0.01Cu–0.01V–0.005Nb–0.015Co, normalized at 1123 °C for 0.17 hrs, tempered at 823 °C for 0.5 hrs, tested under 238MPa at 873 °C	2.4643	−2.5446
Experimental Value of log(tr) = 2.442		
Percentage Error = 0.91%		

Change in an input variable is highlighted in red font to clearly indicate the modified parameter.

## Data Availability

The original contributions presented in this study are included in the article. Further inquiries can be directed to the corresponding authors.

## Abbreviations

The following abbreviations are used in this manuscript:

ANN	Artificial Neural Network
RMSE	Root Mean Squared Error
MSE	Mean Squared Error
MAPE	Mean Absolute Percentage Error
